# High neutrophil-to-lymphocyte ratio predicts short survival in multiple system atrophy

**DOI:** 10.1038/s41531-021-00267-7

**Published:** 2022-01-20

**Authors:** LingYu Zhang, Bei Cao, Yanbing Hou, Qianqian Wei, RuWei Ou, Bi Zhao, Huifang Shang

**Affiliations:** Department of Neurology, Laboratory of Neurodegenerative Disorders, Rare Diseases Center, National Clinical Research Center for Geriatrics, West China Hospital, Sichuan University, Chengdu, China

**Keywords:** Movement disorders, Outcomes research

## Abstract

The neutrophil-to-lymphocyte ratio (NLR), an inflammatory marker, can predict the prognosis of neurodegenerative diseases. However, the significance of NLR for the prognosis of multiple system atrophy (MSA) has not been reported. We aimed to examine the prognostic significance of NLR in MSA. A total of 169 MSA patients and 163 matched healthy controls (HCs) were enrolled. MSA patients were divided into three groups according to the tertiles of their NLR. Kaplan–Meier survival analysis and Cox regression model were used to assessing the effect of NLR on survival. An independent validation cohort of 56 consecutive patients with probable MSA who met the inclusion criteria was included. The NLR was significantly higher in patients with MSA than that in HCs. The survival duration in patients with MSA in group 3 was shorter than that in patients in the other two groups (*P* = 0.013). In the multivariable Cox regression model, a higher NLR increased the risk of mortality in patients with MSA after adjusting for confounding factors (HR = 1.922, *P* = 0.035). Additionally, a higher NLR increased the risk of mortality in MSA with predominant cerebellar ataxia (MSA-C) (HR = 2.398, *P* = 0.033) and in men (HR = 3.483, *P* = 0.027). The concordance index for the multivariate Cox regression model was more than 0.7 both in the primary cohort and external validation cohort. Patients with MSA had a higher NLR than did HCs. A high NLR increased the risk of mortality with MSA, especially in MSA-C and in men.

## Introduction

Multiple system atrophy (MSA) is a severe, progressive neurodegenerative disease that is clinically characterized by varying degrees of parkinsonism, cerebellar ataxia, dysautonomia, and pyramidal features^1^. According to the predominant symptomatology, MSA can be differentiated into two subtypes: parkinsonian (MSA-P) and cerebellar (MSA-C)^2^. No treatment is currently available to modify disease progression in patients with MSA.

The pathological features of MSA include widespread neuronal loss in the basal ganglia, cerebellum, pons, inferior olivary nuclei, and spinal cord, accompanied by gliosis^1^. This correlates with the heterogeneity of the clinical presentation of MSA. The pathological hallmark of MSA is abundant argyrophilic filamentous glial cytoplasmic inclusions (GCIs)^1^. α-Synuclein is the major component of GCIs, and α-synucleinopathy is classified as MSA, Parkinson’s disease (PD), and dementia with Lewy bodies (DLB)^3^. Additionally, several hypotheses regarding the pathogenesis of MSA have been proposed. However, the pathogenic mechanisms are not completely understood. Propagation of misfolded α-synuclein from neurons to oligodendroglia and cell-to-cell spreading, oxidative stress, mitochondrial dysfunction, neuroinflammation, and energy failure are potential pathogenic mechanisms^4^.

Neuroinflammation mediated by astrocytes and microglia is implicated in the pathophysiology of MSA^5^. Activated microglia, as well as GCIs, have been noted in motor-related structures in the post-mortem brain tissue of MSA patients^6^. Additionally, recent studies have reported the immunotherapeutic effects of neurodegenerative disorders, with special emphasis on α-synucleinopathies, including MSA^7^.

The blood neutrophil-to-lymphocyte ratio (NLR) is an inexpensive, easily applicable marker that indicates peripheral inflammation. It is calculated by dividing the absolute number of neutrophils by the absolute number of lymphocytes obtained from the complete blood count. NLR is a predictive factor for the prognosis of cardiovascular disease^8^, cerebrovascular disease^9^, and amyotrophic lateral sclerosis (ALS)^10^. An increasing number of studies have focused on the potential predictors of survival in patients with MSA, including autonomic onset, older age of onset, the higher unified multiple system atrophy rating scale (UMSARS) score, and Parkinsonian variant of MSA^11–14^. However, no studies have focused on the relationship between the NLR and the prognosis in MSA. Therefore, to explore the prognostic significance of NLR in MSA, we evaluated this marker in a large cohort of patients in the early disease stages of MSA.

## Results

### Baseline clinical characteristics

**Fig. 1:**
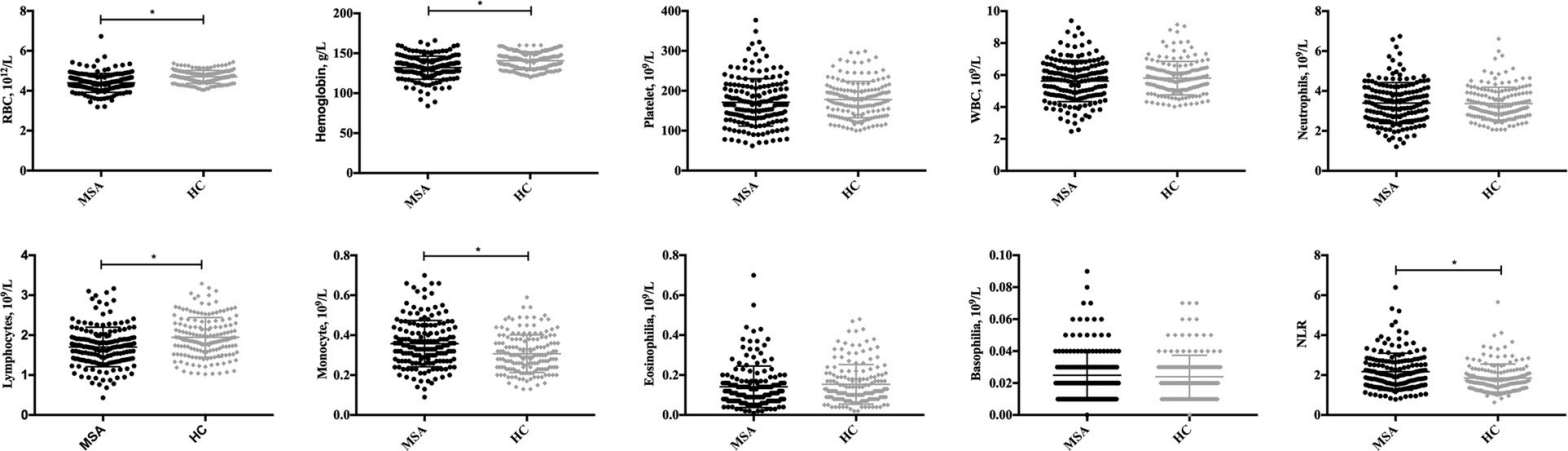
Comparison of hematological data between MSA and HCs. The numbers of patients with MSA and HC were 169 and 163, respectively. The Mann–Whitney *U* test or Student’s *t*-test was performed. The *p*-value of the comparison of RBCs, hemoglobin, lymphocytes, and monocytes between MSA and HCs was <0.001. The *p*-value of the comparison of NLR between MSA and HCs was 0.001. MSA multiple system atrophy, HCs healthy controls. *Indicates significant differences after adjusting for a false discovery rate.

A total of 169 patients with MSA were enrolled in the final analysis at the end of the follow-up. A total of 163 HCs was also evaluated. The demographic and hematological data of the patients with MSA and HCs are shown in Table 1. Age, sex, and BMI were not significantly different between patients with MSA and HCs. Patients with MSA had lower levels of RBCs (4.40 ± 0.47 vs. 4.70 ± 0.33 10^12^/L, *P* < 0.001), hemoglobin (132.04 ± 14.46 vs. 140.80 ± 11.02 g/L, *P* < 0.001), and lymphocytes (1.70 ± 0.49 vs. 1.94 ± 0.50 10^9^/L, *P* < 0.001). These patients had higher monocyte levels (0.36 ± 0.15 vs. 0.31 ± 0.09 10^9^/L, *P* < 0.001) and NLR (2.17 ± 0.92 vs. 1.85 ± 0.70, *P* = 0.001) than did HCs (Fig. 1). For the validation cohort, the demographic and hematological data of the 56 patients with MSA are shown in Supplementary Table 2.

**Table 1:** Demographic and the hematological data of MSA and HC.

Variables	Total MSA	HC	*P*-value
Number	169	163	–
Age (yr)	59.02 ± 7.93	59.54 ± 9.21	0.579
Sex (male, %)	43.8	49.3	0.944
BMI	23.33 ± 3.27	23.81 ± 2.09	0.114
RBCs (10^12^/L)	4.40 ± 0.47	4.70 ± 0.33	<0.001*
Hemoglobin (g/L)	132.04 ± 14.46	140.80 ± 11.02	<0.001*
Platelet (10^9^/L)	171.21 ± 59.43	178.03 ± 45.38	0.242
WBCs (10^9^/L)	5.68 ± 1.47	5.80 ± 1.05	0.395
Neutrophils (10^9^/L)	3.44 ± 1.19	3.37 ± 0.82	0.544
Lymphocytes (10^9^/L)	1.70 ± 0.49	1.94 ± 0.50	<0.001*
Monocyte (10^9^/L)	0.36 ± 0.15	0.31 ± 0.09	<0.001*
Eosinophilia (10^9^/L)	0.15 ± 0.14	0.15 ± 0.10	0.937
Basophilia (10^9^/L)	0.03 ± 0.02	0.02 ± 0.01	0.350
NLR	2.17 ± 0.92	1.85 ± 0.70	0.001*

*MSA* multiple system atrophy, *HC* healthy control, *BMI* body mass index, *RBCs* red blood cells, *WBCs* white blood cells, *NLR* neutrophil-to-lymphocyte ratio.

*Significant difference after adjusting by false discovery rate.

### Clinical and hematological data of MSA patients in different groups

The demographic and clinical features of patients with MSA in the three groups according to NLR are shown in Table 2. The mean age and age at onset of patients were 59.02 ± 7.93 and 57.56 ± 7.82 years, respectively. Among them, 32% of the patients with MSA-P and 43.8% of the men were enrolled in the cohort. The mean disease duration was 1.82 ± 0.68 years at baseline. There were no significant differences in age, subtype, sex, disease duration, OH, UMSARS-I, UMSARS-II, UMSARS-IV, total UMSARS score, history of smoking and drinking, comorbidities, or treatments between the three groups. Additionally, we found that NLR was not associated with disease progression of MSA (*P* > 0.05), according to Spearman’s correlation analysis (Supplementary Table 1).

**Table 2:** Demographic and clinical features of patients with MSA between the three groups according to NLR tertiles.

Variables	Total MSA	Group 1	Group 2	Group 3	*P* value^a^
Number	169	56	57	56	–
Diagnosis subtype (MSA-P, %)	32.0	28.6	36.8	30.4	0.611
Age (yr)	59.02 ± 7.93	59.56 ± 7.88	59.42 ± 8.09	58.05 ± 7.86	0.537
Age of onset (yr)	57.56 ± 7.82	57.88 ± 7.68	57.90 ± 8.06	56.88 ± 7.81	0.735
Sex (male, %)	43.8	41.1	45.6	44.6	0.877
BMI	23.33 ± 3.27	23.60 ± 3.32	23.52 ± 3.35	22.88 ± 3.16	0.452
Disease duration (yr)	1.82 ± 0.68	1.81 ± 0.71	1.91 ± 0.68	1.74 ± 0.66	0.399
Onset symptom (motor onset, %)	52.7%	64.3%	43.9%	50.0%	0.083
UMSARS-I	16.37 ± 6.72	15.93 ± 6.31	16.56 ± 7.17	16.63 ± 6.75	0.834
UMSARS-II	18.04 ± 7.81	18.43 ± 7.18	17.79 ± 7.86	17.91 ± 8.48	0.900
UMSARS-IV	2.05 ± 0.97	2.04 ± 0.93	2.05 ± 1.03	2.07 ± 0.97	0.981
Total UMSARS score	34.41 ± 13.96	34.36 ± 12.76	34.35 ± 14.46	34.54 ± 14.82	0.997
OH (%)	24.9	17.9	22.8	33.9	0.131
Smoking (%)	45 (26.6%)	14 (25.0%)	13 (22.8%)	18 (32.1%)	0.503
Drinking (%)	39 (23.1%)	13 (23.2%)	10 (17.5%)	16 (28.6%)	0.380
*Comorbidity*					
Hypertension (%)	23 (13.6%)	11 (19.6%)	6 (10.5%)	6 (10.7%)	0.273
Diabetes mellitus (%)	17 (10.1%)	4 (7.1%)	9 (15.8%)	4 (7.1%)	0.210
Hyperlipidemia (%)	22 (13.0%)	6 (10.7%)	9 (15.8%)	7 (12.5%)	0.718
*Treatment*					
Levodopa (%)	22 (13.0%)	6 (10.7%)	8 (14.0%)	8 (14.3%)	0.821
Amantadine (%)	7 (4.1%)	2 (3.6%)	2 (3.5%)	3 (5.4%)	0.856
Dopamine receptor agonist (%)	5 (3.0%)	3 (5.4%)	2 (3.5%)	0 (0.0%)	0.115
Buspirone hydrochloride (%)	7 (4.1%)	2 (3.6%)	3 (5.3%)	2 (3.6%)	0.873
Hypotensive drug (%)	23 (13.6%)	11 (19.6%)	6 (10.5%)	6 (10.7%)	0.273
Hypoglycemic agent (%)	17 (10.1%)	4 (7.1%)	9 (15.8%)	4 (7.1%)	0.210
lipid-lowering drug (%)	15 (8.9%)	4 (7.1%)	6 (10.5%)	5 (8.9%)	0.819

*MSA* multiple system atrophy, *NLR* neutrophil-to-lymphocyte ratio, *MSA-P* multiple system atrophy with predominate parkinsonism, *BMI* body mass index, *UMSARS* unified multiple system atrophy rating scale, *OH* orthostatic hypotension.

^a^Compared among groups 1, 2, and 3.

Table 3 shows the hematological data of patients with MSA in the three groups. The levels of RBCs, WBCs, neutrophils, and lymphocytes were significantly different among the three groups after adjusting for FDR. Group 3 had the highest levels of RBCs (4.53 ± 0.51 × 10^12^/L), WBCs (6.16 ± 1.73 × 10^9^/L), and neutrophils (4.25 ± 1.33 × 10^9^/L) compared to the other two groups. Group 3 had the lowest levels of lymphocytes (1.36 ± 0.33 × 10^9^/L) compared to the other two groups. There were no significant differences in the levels of hemoglobin, platelets, monocytes, eosinophilia, or basophilia between the three groups.

**Table 3:** Hematological data of patients with MSA between the three groups according to NLR tertiles.

Hematological tests	Total MSA	Group 1	Group 2	Group 3	*P* value^a^
RBCs (10^12^/L)	4.40 ± 0.47	4.32 ± 0.38	4.34 ± 0.50	4.53 ± 0.51	0.023
Hemoglobin (g/L)	132.04 ± 14.46	130.39 ± 14.64	130.72 ± 11.17	135.02 ± 16.84	0.121
Platelet (10^9^/L)	171.21 ± 59.43	170.88 ± 52.54	180.04 ± 65.35	162.55 ± 59.35	0.448
WBCs (10^9^/L)	5.68 ± 1.47	5.23 ± 1.10	5.66 ± 1.38	6.16 ± 1.73	0.004*
Neutrophils (10^9^/L)	3.44 ± 1.19	2.65 ± 0.65	3.42 ± 0.91	4.25 ± 1.33	<0.001*
Lymphocytes (10^9^/L)	1.70 ± 0.49	2.06 ± 0.48	1.70 ± 0.38	1.36 ± 0.33	<0.001*
Monocyte (10^9^/L)	0.36 ± 0.15	0.34 ± 0.10	0.36 ± 0.12	0.40 ± 0.20	0.266
Eosinophilia (10^9^/L)	0.15 ± 0.14	0.16 ± 0.16	0.16 ± 0.11	0.14 ± 0.16	0.216
Basophilia (10^9^/L)	0.03 ± 0.02	0.03 ± 0.02	0.02 ± 0.01	0.02 ± 0.01	0.070
NLR	2.17 ± 0.92	1.31 ± 0.22	2.00 ± 0.20	3.20 ± 0.81	<0.001*

*MSA* multiple system atrophy, *NLR* neutrophil-to-lymphocyte ratio, *RBCs* red blood cells, *WBCs* white blood cells.

*Significant difference after adjusting by false discovery rate.

^a^Compared among groups 1, 2, and 3.

### Survival analysis of NLR in patients with MSA

**Fig. 2:**
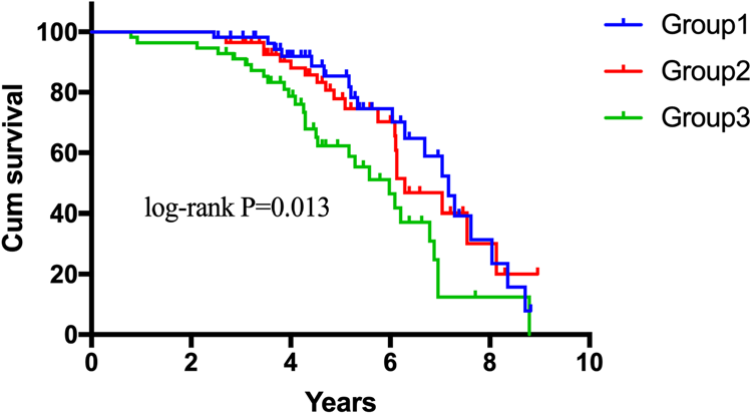
Comparison of survival between the three groups according to NLR tertiles. The numbers of group 1, group 2, and group 3 were 56, 57, and 56, respectively. Kaplan–Meier survival analysis and log-rank tests were performed (log-rank *p* = 0.013). NLR: neutrophil-to-lymphocyte ratio.

Sixty-seven (39.6%) patients with MSA died, and 102 (60.4%) were alive at the end of the follow-up period. The mean survival duration of all MSA patients and deceased patients was 6.3 and 5.1 years, respectively. As shown in Fig. 2, the survival duration was significantly different among the three groups (log-rank *P* = 0.013). The survival duration of patients in group 3 was shorter than those in group 2 (estimated mean survival time: 5.6 vs. 6.6 years, log-rank *P* = 0.029) and group 1 (estimated mean survival time: 5.6 vs. 6.8 years, log-rank *P* = 0.007).

Univariate Cox proportional hazards regression analysis for survival is shown in Table 4. Higher levels of WBC (HR = 1.239, 95% CI = 1.051–1.459, *P* = 0.010), neutrophils (HR = 1.357, 95% CI = 1.131–1.628, *P* = 0.001), and NLR (HR = 1.412, 95% CI = 1.098–1.816, *P* = 0.007) were associated with an increased risk of mortality in patients with MSA than in HCs.

**Table 4:** Univariate Cox proportional-hazards regression analyses for survival.

Hematological tests	HR (95% CI)	*P* value
RBCs	0.981 (0.551–1.747)	0.948
Hemoglobin	1.001 (0.982–1.021)	0.891
Platelet	0.999 (0.994–1.003)	0.620
WBCs	1.239 (1.051–1.459)	0.010*
Neutrophils	1.357 (1.131–1.628)	0.001*
Lymphocytes	0.773 (0.470–1.273)	0.312
Monocyte	4.017 (0.946–17.059)	0.060
Eosinophilia	3.990 (0.826–19.277)	0.085
Basophilia	42.255 (0.000–5111.6)	0.600
NLR	1.412 (1.098–1.816)	0.007*

*RBCs* red blood cells, *WBCs* white blood cells, *NLR* neutrophil-to-lymphocyte ratio.

*Significant difference after adjusting by false discovery rate.

To further demonstrate the predictive significance of NLR on survival, we performed a multivariable Cox proportional hazards regression analysis (Table 5). After adjusting for age, sex, subtype, symptom onset, BMI, disease duration, total UMSARS score, OH, and urinary incontinence, a higher NLR (continuous variable) was associated with an increased risk of mortality in patients with MSA (HR = 1.404, 95% CI 1.027–1.919, *P* = 0.034). When NLR was divided into three levels according to NLR tertiles, the highest NLR increased the risk of mortality in patients with MSA after adjusting for age, sex, subtype, symptom onset, BMI, disease duration, total UMSARS score, OH, and urinary incontinence (HR = 1.922, 95% CI, 1.046–3.531; *P* = 0.035). Additionally, the highest NLR increased the risk of mortality in patients with MSA-C after adjusting for age, sex, symptom onset, BMI, disease duration, total UMSARS score, OH, and urinary incontinence (HR = 2.398, 95% CI 1.073–5.359, *P* = 0.033). The highest NLR increased the risk of mortality in men after adjusting for age, subtype, symptom onset, BMI, disease duration, total UMSARS score, OH, and urinary incontinence (HR = 3.483, 95% CI 1.149–10.556, *P* = 0.027).

**Table 5:** Multivariate Cox proportional-hazards regression analyses for survival.

Variables	HR (95% CI)	*P* value
NLR (continuous)^a^	1.404 (1.027–1.919)	0.034*
NLR (categorized)^b^	1.000 (Ref)	
	1.102 (0.575–2.113)	0.769
	1.922 (1.046–3.531)	0.035*
	*P* trend	0.066
NLR (categorized)^c^	1.000 (Ref)	
	0.740 (0.309–1.774)	0.500
	2.398 (1.073–5.359)	0.033*
	*P* trend	0.013*
NLR (categorized)^d^	1.000 (Ref)	
	0.563(0.175–1.816)	0.336
	3.483 (1.149–10.556)	0.027*
	*P* trend	0.003*

*NLR* neutrophil-to-lymphocyte ratio.

*Significant difference.

^a^Adjusting for age, sex, subtype, onset symptom, BMI, disease duration, total UMSARS score, OH, and urinary incontinence.

^b^Adjusting for age, sex, subtype, onset symptom, BMI, disease duration, total UMSARS score, OH, and urinary incontinence.

^c^In MSA-C patients, adjusting for age, sex, onset symptom, BMI, disease duration, total UMSARS score, OH, and urinary incontinence.

^d^In male patients, adjusting for age, subtype, onset symptom, BMI, disease duration, total UMSARS score, OH, and urinary incontinence.

### The validation of survival analysis in patients with MSA

In the independent validation cohort, multivariate Cox proportional hazards regression analysis showed that higher NLR (continuous variable) or the highest NLR (categorized variable) were associated with an increased risk of mortality in patients with MSA (HR = 1.963, 95% CI 1.389–2.773, *P* < 0.001, HR = 12.435, 95% CI 1.359–113.733, *P* = 0.026, respectively), after adjusting for age, sex, subtype, symptom onset, BMI, disease duration, total UMSARS score, OH, and urinary incontinence (Supplementary Table 3).

Additionally, the C-index for this multivariate Cox proportional-hazards regression model was 0.724 (NLR as a continuous variable) and 0.723 (NLR as a categorical variable) in the primary cohort. The C-index in the external validation cohort was 0.855 (NLR as a continuous variable) and 0.785 (NLR as a categorical variable).

## Discussion

To the best of our knowledge, this is the first study to explore the significance of NLR at the first evaluation of survival in a large cohort of patients with early-phase MSA (disease duration < 3 years from the first onset of symptoms). In this study, patients with MSA had a higher NLR than HCs. A high NLR was significantly associated with poor survival in patients with MSA, especially in the MSA-C subtype and in men.

Additionally, we found that NLR was not associated with disease progression in 31 patients with MSA, possibly owing to the small sample size. A longitudinal study testing NLR at various time points will assist us in further assessing the role of NLR in the progression of MSA. The C-index of the multivariate Cox regression model indicated that the prognostic prediction model was reliable.

We found that the NLR was significantly higher in patients with MSA than in HCs. NLR has been reported to be higher in patients with Alzheimer’s disease (AD) and PD than in HCs^15,16^. Recently, a meta-analysis showed that the NLR was higher in PD patients than in HCs^17^, which supports our results as PD and MSA are α-synucleinopathies.

NLR is a noninvasive and an excellent general indicator of systemic inflammation. Systemic inflammation contributes to the symptoms and progression of neurodegenerative diseases^18^. The relationship between inflammation and MSA has been demonstrated in previous studies^19–21^. The evidence suggested that rs1799964 of *TNF-α* related to inflammation may act as a risk factor for MSA^19^. Kaufman et al. reported that the levels of IL-6 and TNF-α were significantly more elevated in patients with MSA than in HCs. This suggests a link between peripheral inflammation and MSA^21^. In addition, it has been reported that patients with MSA display evidence of increased markers of endotoxin-related intestinal inflammation and pro-inflammatory colonic microbiota^20^. This evidence indicates that inflammation plays a critical role in MSA pathogenesis. Neutrophils have been reported to play an important role in chronic inflammation^22^. Although migration of neutrophils into the infected tissue is one of the features of acute inflammation, neutrophils can be replaced by macrophages and lymphocytes^23^. Macrophages, lymphocytes, and plasma cells at the tissue site are responsible for chronic inflammation^23^. The increased absolute neutrophil count in MSA could indicate that chronic inflammation exists in the early stages of MSA. The decreased absolute lymphocyte count in MSA patients could reflect a loss of the protective immune function of lymphocytes in MSA, as has been reported in PD^24^.

Moreover, the NLR was not correlated with age, disease duration, OH, UMSARS-I, UMSARS-II, UMSARS-IV, or total UMSARS score. These results suggest that the NLR is independent of age, disease duration, and disease severity in the early stage of the disease. This indicates that NLR is a good indicator of peripheral inflammation status and immune dysregulation in MSA. The multivariable Cox regression analysis showed that a higher NLR (continuous or categorized variable) was associated with a significantly increased risk of mortality in patients with MSA. The prognostic value of NLR was also demonstrated in the MSA-C subtype and in men. NLR has been reported to be a significant predictor of shorter survival in patients with cardiovascular disease^8^, cerebrovascular disease^9^, and ALS^10^.

To the best of our knowledge, this is the first study to examine NLR as an inflammatory biomarker for survival in patients with MSA. Sex affects the various steps of an immune response based on hormonal and genetic effects. Overall, estrogen can enhance immune responses, whereas androgens can decrease it^25^. In addition, the X chromosome can regulate the immune response^25^. This evidence might partially explain the higher NLR associated with a significantly increased risk of mortality in male patients with MSA. However, the mechanism of the different prognoses based on the NLR between MSA-P and MSA-C is unclear. Further research is required to clarify this mechanism. Additionally, our results indicate that NLR may be a useful prognostic biomarker in patients with MSA. This is the first study to demonstrate that a higher NLR predicts shorter survival in patients with MSA. However, it lacks evidence regarding the management of NLR. Nevertheless, clinicians should focus on comprehensive management to extend the survival duration in patients with a higher NLR. This management includes drug treatment combined with rehabilitation exercises, blood pressure monitoring, prevention of urinary tract infections, and avoidance of falls.

There were several limitations to the current study. First, we omitted other peripheral inflammatory biomarkers. Second, this study was conducted in a single cohort at a single hospital. Third, we only estimated the association between the baseline NLR and survival in patients with MSA. Thus, a longitudinal study examining NLR at various time points will help us further assess the role of NLR in the progression of MSA. We will address this issue in a future large cohort study aimed at exploring whether NLR changes during disease progression. Fourth, all patients were clinically diagnosed without a postmortem diagnosis.

This is the first study to explore the potential predictive significance of NLR on survival in patients with early-phase MSA. Patients with MSA had a higher NLR than did HCs. Additionally, we found that a high NLR increased the risk of mortality in patients with MSA, especially in those with MSA-C and in men.

## Methods

### Study design and participants

**Fig. 3:**
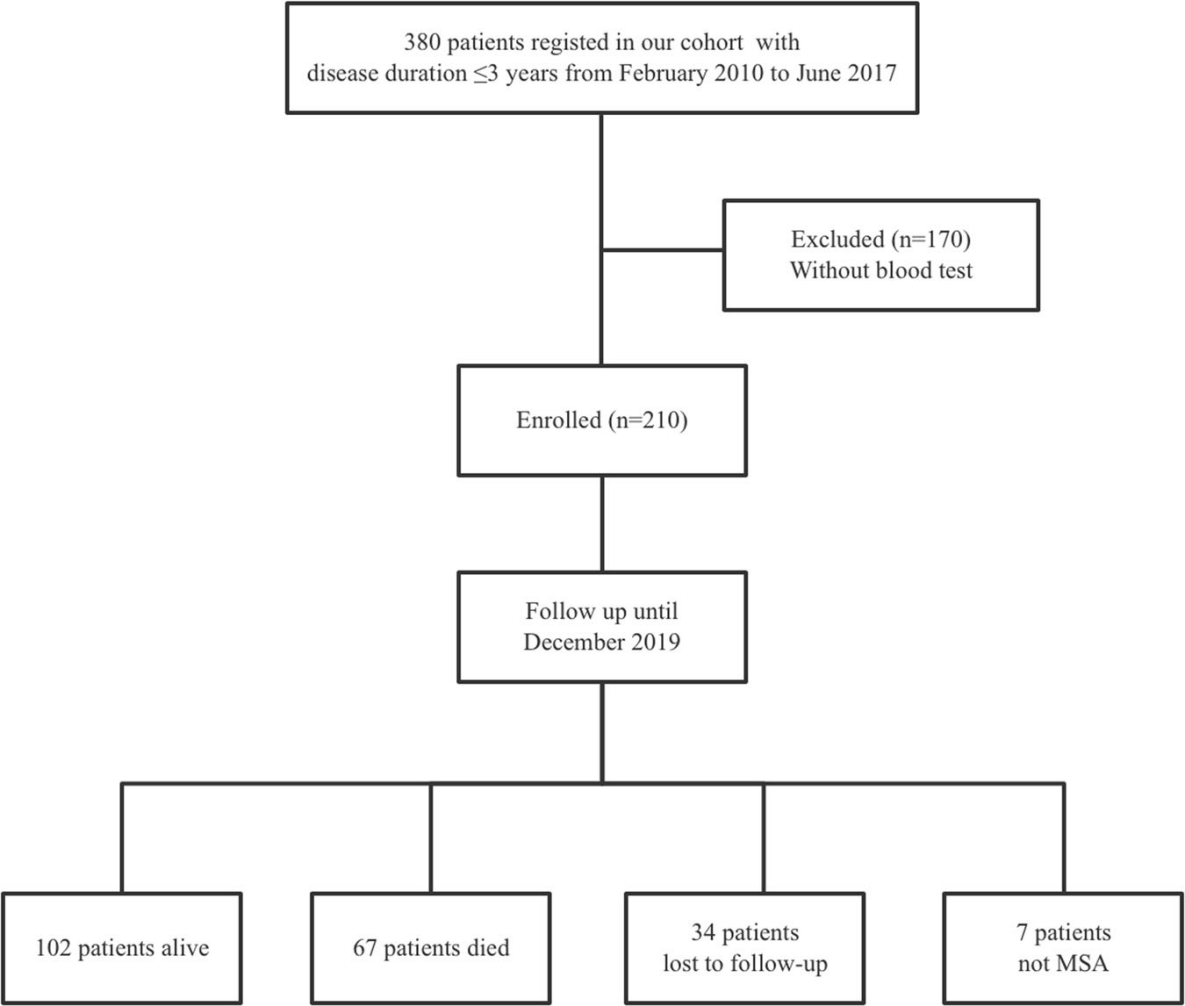
Study flow diagram. MSA multiple system atrophy.

A total of 380 patients diagnosed with early phase MSA (disease duration <3 years) at the Department of Neurology, West China Hospital of Sichuan University, between February 2010 and June 2017, were recruited for the study. Patients with urinary tract infections, bronchopneumonia, or aspiration pneumonia at the initial assessment were excluded. All patients fulfilled the possible or probable MSA clinical diagnostic criteria^2^. Patients were screened for spinocerebellar ataxia (SCA) genes, including SCA1, -2, -3, -6, and -7, to exclude the common forms of SCA. They also received brain MRI scans to exclude other neurological disorders. Patients were excluded if a blood test was not performed within 3 months of the initial clinical assessment (*N* = 170). Furthermore, 210 patients were followed every year by neurologists via telephone or in-person interviews. During follow-up appointments, patients diagnosed with PD (*n* = 3), progressive supranuclear palsy (PSP) (*n* = 3), and cortical basal degeneration (*n* = 1) were excluded. Thirty-four patients were lost to follow-up. Finally, 169 patients with a probable diagnosis of MSA were included in the final analysis (Fig. 3). Additionally, 163 age-, sex-, and body mass index (BMI)-matched healthy controls (HCs) were enrolled. Moreover, we included 31 patients with a follow-up period of one year and a UMSARS assessment at the one-year follow-up appointment. An independent validation cohort of 56 consecutive patients with probable MSA who met the inclusion criteria was included from July 2017 to February 2019. All patients were followed every year by neurologists via telephone or in-person interviews until July 2021.

### Demographic and clinical data collection

According to the predominant parkinsonian features or cerebellar ataxia, patients with MSA were divided into two subtypes at the time of evaluation: MSA-P or MSA-C. All patients were evaluated during in-person interviews with neurologists. Clinical information including sex, age, weight, height, age at onset, symptom onset, disease duration, years of education, history of smoking and drinking, comorbidities, and treatments were collected. Disease duration was defined as the time from the date of disease onset to the date of evaluation. Symptom onset was defined as the initial presentation of any motor symptoms (i.e., parkinsonism or cerebellar ataxia) or autonomic features, with the exception of erectile dysfunction^2^. Survival duration was defined as the interval from the date of disease onset to the date of death for a deceased patient or from disease onset to the last follow-up appointment for surviving patients. Death information was collected from the provincial public security bureau records and family reports.

### Evaluation protocol

Disease severity was rated on part I (activities of daily living, ADL), part II (motor examination), part III (autonomic examination), and part IV (global disability) of the UMSARS^26^. The total UMSARS score is the sum of parts I and II. Orthostatic hypotension (OH) was defined as a drop in SBP of ≥30 mmHg and/or DBP of ≥15 mmHg. First, the patients laid down on the examination table for 10 min before their supine blood pressure (BP) was measured. Further, the patients were asked to stand up and their BP was recorded at 1, 3, 5, and 10 min. BMI was calculated as body weight (kg) divided by height squared (m^2^).

### Hematological data collection

Blood sampling was performed after overnight fasting during the first assessment within 3 months. The following hematological tests were considered for the study: white blood cell (WBC) count (neutrophil, lymphocyte, monocyte, eosinophilia, and basophilia), red blood cell (RBC) count, hemoglobin, and platelet count.

Informed written consent was obtained from all participants. The study design was approved by the Ethics Committee of West China Hospital of Sichuan University.

### Statistical analysis

Patients were divided into three groups (group 1: <1.64, group 2: 1.64–2.38, and group 3: >2.38) according to NLR tertiles^10^. All continuous data are presented as the mean ± standard deviation. All categorical variables are presented as numbers or percentages. For each continuous variable, we used the Kolmogorov–Smirnov test to evaluate the normality of the distribution. An analysis of variance (ANOVA) test or Kruskal–Wallis test was used to compare continuous variables between different groups regarding NLR. The Mann–Whitney *U* test or Student’s *t*-test was used to comparing continuous variables between the MSA and HC groups. A chi-square test was performed to compare categorical variables (*P-value* was adjusted by false discovery rate [FDR]). The annual progression rate of UMSARS total score = (1-year follow-up UMSARS total score−baseline UMSARS total score)/year, represented the disease progression rate. Spearman’s correlation was performed to assess the association between NLR and disease progression. Kaplan–Meier survival analysis and log-rank test were used to analyze the prognostic significance of the NLR for survival. Univariate and multivariate survival analyses were performed using the Cox proportional hazards regression model. In the multivariate Cox proportional hazards regression model, we adjusted for age (continuous variable), sex (male vs. female), subtype (MSA-P vs. MSA-C), symptom onset (motor onset vs. autonomic onset), BMI (continuous variable), disease duration (continuous variable, in years), total UMSARS score (continuous variable), OH (yes vs. no), and urinary incontinence (yes vs. no). The concordance index (C-index) was used to evaluate the accuracy of the prognostic prediction model.

Statistical significance was set at *P* < 0.05. IBM SPSS software (version 22.0) and R version 3.6.3 were used for the statistical analysis.

### Reporting summary

Further information on research design is available in the Nature Research Reporting Summary linked to this article.

## Supplementary information

Supplementary Information

Reporting Summary

## Data Availability

The authors confirm that the data supporting the findings of this study are available within the article.
