# Supplementary material for: High neutrophil-to-lymphocyte ratio predicts short survival in multiple system atrophy
Source: NPJ Parkinsons Dis. 2022 Jan 20;8:11. doi: 10.1038/s41531-021-00267-7 (PMC8776861; doi:10.1038/s41531-021-00267-7)
Supplement: Supplementary file 1 — Supplementary Information [file 41531_2021_267_MOESM1_ESM.pdf]

Supplementary Table 1 Correlation between NLR and disease progression in 31 patients with MSA.

Variables	Annual progression rate of UMSARS total score	
	Correlation coefficient	P value
NLR (continuous)	0.118	0.127
NLR (categorized)	0.057	0.465

NLR: neutrophil-to-lymphocyte ratio.

Supplementary Table 2 Demographic and hematological data of 56 patients with MSA.

Variables	Total MSA
Number	56
Diagnosis subtype (MSA-P, %)	22 (39.3%)
Age (y)	59.19±8.02
Age of onset (y)	57.34±7.94
Sex (male, %)	29 (51.8%)
BMI	23.50±3.22
Disease duration (y)	1.80±0.69
Onset symptom (motor onset, %)	33 (58.9%)
UMSARS-I	17.25±5.96
UMSARS-II	19.54±7.66
UMSARS-IV	2.20±0.90
Total UMSARS score	36.79±12.57
OH (%)	25 (44.6%)
RBCs (10^{12}/L)	4.53±0.47
Hemoglobin (g/L)	135.07±15.48
Platelet (10^9/L)	185.32±58.90
WBCs (10^9/L)	5.97±2.13
Neutrophils (10^9/L)	3.53±1.40
Lymphocytes (10^9/L)	1.76±0.55
Monocyte (10^9/L)	0.38±0.16
Eosinophilia (10^9/L)	0.18±0.26
Basophilia (10^9/L)	0.03±0.02
NLR	2.18±1.22

MSA: multiple system atrophy; MSA-P: multiple system atrophy with predominate parkinsonism; BMI: body mass index; UMSARS, unified multiple system atrophy rating scale; OH, orthostatic hypotension; RBCs: red blood cells; WBCs: white blood cells; NLR: neutrophil-to-lymphocyte ratio.

Supplementary Table 3 Multivariate Cox proportional-hazards regression analysis for survival.

Variables	HR (95%CI)	P value
NLR (continuous)[a]	1.963 (1.389-2.773)	<0.001*
NLR (categorized)[a]	1.000 (Ref)	
	6.109 (0.744-50.178)	0.092
	12.435 (1.359-113.733)	0.026*
	P trend	0.074

NLR: neutrophil-to-lymphocyte ratio.

[a] adjusting age, sex, subtype, onset symptom, BMI, disease duration, total UMSARS score, OH, and urinary incontinence.

*significant difference.
