# Supplementary material for: High neutrophil-to-lymphocyte ratio predicts short survival in multiple system atrophy
Source: NPJ Parkinsons Dis. 2022 Jan 20;8:11. doi: 10.1038/s41531-021-00267-7 (PMC8776861; doi:10.1038/s41531-021-00267-7)
Supplement: Supplementary file 2 — Reporting Summary [file 41531_2021_267_MOESM2_ESM.pdf]

nature portfolio

Corresponding author(s): Huifang Shang

Last updated by author(s): Dec 8, 2021

# Reporting Summary

Nature Portfolio wishes to improve the reproducibility of the work that we publish. This form provides structure for consistency and transparency in reporting. For further information on Nature Portfolio policies, see our Editorial Policies and the Editorial Policy Checklist.

## Statistics

For all statistical analyses, confirm that the following items are present in the figure legend, table legend, main text, or Methods section.

| n/a | Confirmed | |
|---|---|---|
| ☐ | ☒ | The exact sample size ($n$) for each experimental group/condition, given as a discrete number and unit of measurement |
| ☒ | ☐ | A statement on whether measurements were taken from distinct samples or whether the same sample was measured repeatedly |
| ☐ | ☒ | The statistical test(s) used AND whether they are one- or two-sided<br>*Only common tests should be described solely by name; describe more complex techniques in the Methods section.* |
| ☐ | ☒ | A description of all covariates tested |
| ☐ | ☒ | A description of any assumptions or corrections, such as tests of normality and adjustment for multiple comparisons |
| ☐ | ☒ | A full description of the statistical parameters including central tendency (e.g. means) or other basic estimates (e.g. regression coefficient) AND variation (e.g. standard deviation) or associated estimates of uncertainty (e.g. confidence intervals) |
| ☒ | ☐ | For null hypothesis testing, the test statistic (e.g. $F$, $t$, $r$) with confidence intervals, effect sizes, degrees of freedom and $P$ value noted<br>*Give P values as exact values whenever suitable.* |
| ☒ | ☐ | For Bayesian analysis, information on the choice of priors and Markov chain Monte Carlo settings |
| ☒ | ☐ | For hierarchical and complex designs, identification of the appropriate level for tests and full reporting of outcomes |
| ☒ | ☐ | Estimates of effect sizes (e.g. Cohen's $d$, Pearson's $r$), indicating how they were calculated |

*Our web collection on statistics for biologists contains articles on many of the points above.*

## Software and code

Policy information about availability of computer code

| | |
|---|---|
| Data collection | No software was used. |
| Data analysis | IBM SPSS software (version 22.0) and R version 3.6.3 were used for the statistical analysis. |

For manuscripts utilizing custom algorithms or software that are central to the research but not yet described in published literature, software must be made available to editors and reviewers. We strongly encourage code deposition in a community repository (e.g. GitHub). See the Nature Portfolio guidelines for submitting code & software for further information.

## Data

Policy information about availability of data

All manuscripts must include a data availability statement. This statement should provide the following information, where applicable:

- Accession codes, unique identifiers, or web links for publicly available datasets
- A description of any restrictions on data availability
- For clinical datasets or third party data, please ensure that the statement adheres to our policy

The authors confirm that the data supporting the findings of this study are available within the article.

# Field-specific reporting

Please select the one below that is the best fit for your research. If you are not sure, read the appropriate sections before making your selection.

☒ Life sciences ☐ Behavioural & social sciences ☐ Ecological, evolutionary & environmental sciences

For a reference copy of the document with all sections, see nature.com/documents/nr-reporting-summary-flat.pdf

# Life sciences study design

All studies must disclose on these points even when the disclosure is negative.

| | |
|---|---|
| Sample size | 169 patients with a probable diagnosis of MSA were included in the final analysis. An independent validation cohort of 56 consecutive patients with probable MSA who met the inclusion criteria were included from July 2017 to February 2019. |
| Data exclusions | Patients with urinary tract infections, bronchopneumonia, or aspiration pneumonia at the initial assessment were excluded. Patients were excluded if the blood test was not performed within 3 months of the initial clinical assessment (N=170).During follow-up, patients diagnosed with PD (N=3), progressive supranuclear palsy (PSP) (N=3), and cortical basal degeneration (N=1) were excluded. |
| Replication | No replication was performed in the current study. |
| Randomization | No randomization was performed in the current study. |
| Blinding | No blinding was performed in the current study. |

# Reporting for specific materials, systems and methods

We require information from authors about some types of materials, experimental systems and methods used in many studies. Here, indicate whether each material, system or method listed is relevant to your study. If you are not sure if a list item applies to your research, read the appropriate section before selecting a response.

## Materials & experimental systems

| n/a | Involved in the study | |
|---|---|---|
| ☒ | ☐ | Antibodies |
| ☒ | ☐ | Eukaryotic cell lines |
| ☒ | ☐ | Palaeontology and archaeology |
| ☒ | ☐ | Animals and other organisms |
| ☐ | ☒ | Human research participants |
| ☐ | ☒ | Clinical data |
| ☒ | ☐ | Dual use research of concern |

## Methods

| n/a | Involved in the study | |
|---|---|---|
| ☒ | ☐ | ChIP-seq |
| ☒ | ☐ | Flow cytometry |
| ☒ | ☐ | MRI-based neuroimaging |

## Human research participants

Policy information about studies involving human research participants

| | |
|---|---|
| Population characteristics | A total of 169 patients with a probable diagnosis of MSA were included in the final analysis.The mean age and age of onset of patients were 59.02 ± 7.93 and 57.56 ± 7.82 years, respectively. Among which, 32% patients with MSA-P and 43.8% male patients were enrolled in the cohort. The mean disease duration was 1.82 ± 0.68 years at baseline. Furthermore, 163 age-, sex-, and body mass index (BMI)-matched healthy controls (HCs) were enrolled. |
| Recruitment | A total of 380 patients diagnosed with MSA at an early phase (disease duration < 3 years) at the Department of Neurology, West China Hospital of Sichuan University, between February 2010 and June 2017, were recruited in the study. An independent validation cohort of 56 consecutive patients with probable MSA who met the inclusion criteria were included from July 2017 to February 2019. |
| Ethics oversight | Informed written consent was obtained from all participants. The study design was approved by the Ethics Committee of West China Hospital of Sichuan University. |

Note that full information on the approval of the study protocol must also be provided in the manuscript.

## Clinical data

Policy information about clinical studies

All manuscripts should comply with the ICMJE guidelines for publication of clinical research and a completed CONSORT checklist must be included with all submissions.

| | |
|---|---|
| Clinical trial registration | Not applicable. |
| Study protocol | Not applicable. |
| Data collection | All patients were evaluated during in-person interviews with neurologists. |
| Outcomes | Death is the outcome. |
